# Effectiveness and safety of pelareorep plus chemotherapy versus chemotherapy alone for advanced solid tumors: a meta-analysis

**DOI:** 10.3389/fphar.2023.1228225

**Published:** 2023-09-26

**Authors:** Renxian Xie, Hongxin Huang, Tong Chen, Xuehan Huang, Chuangzhen Chen

**Affiliations:** ^1^ Department of Radiation Oncology, Cancer Hospital of Shantou University Medical College, Shantou, China; ^2^ Shantou University Medical College, Shantou, China

**Keywords:** pelareorep, oncolytic virus, chemotherapy, effectiveness, cancer

## Abstract

**Background:** Pelareorep is an oncolytic virus that causes oncolytic effects in many solid tumors, and it has shown therapeutic benefits. However, few studies have compared pelareorep combined with chemotherapy to traditional chemotherapy alone in advanced solid tumors. Consequently, we intended to evaluate the effectiveness and safety of pelareorep plus chemotherapy in this paper.

**Methods:** We searched four databases including PubMed, Embase, Cochrane Library and Web of Science comprehensively for studies comparing pelareorep combined with chemotherapy to chemotherapy alone in the treatment of advanced solid tumors. The outcomes measures were 1-year overall survival (OS), 2-year OS, 4-month progression-free survival (PFS), 1-year PFS, objective response rate (ORR), any-grade adverse events (any-grade AEs), and severe AEs (grade ≥ 3).

**Results:** There were five studies involving 492 patients included in the study. Combination therapy did not significantly improve clinical outcomes in terms of 1-year OS [RR = 1.02, 95%CI = (0.82–1.25)], 2-year OS [RR = 1.00, 95%CI = (0.67–1.49)], 4-month PFS [RR = 1.00, 95%CI = (0.67–1.49)], 1-year PFS [RR = 0.79, 95%CI = (0.44–1.42)], and ORR [OR = 0.79, 95%CI = (0.49–1.27)] compared to chemotherapy alone, and the subgroup analysis of 2-year OS, 1-year PFS, and ORR based on countries and tumor sites showed similar results. In all grades, the incidence of AEs was greater with combination therapy, including fever [RR = 3.10, 95%CI = (1.48–6.52)], nausea [RR = 1.19, 95%CI = (1.02–1.38)], diarrhea [RR = 1.87, 95%CI = (1.39–2.52)], chills [RR = 4.14, 95%CI = (2.30–7.43)], headache [RR = 1.46, 95%CI = (1.02–2.09)], vomiting [RR = 1.38, 95%CI = (1.06–1.80)] and flu-like symptoms [RR = 4.18, 95%CI = (2.19–7.98)]. However, severe adverse events did not differ significantly between the two arms.

**Conclusion:** Pelareorep addition to traditional chemotherapy did not lead to significant improvements in OS, PFS, or ORR in advanced solid tumor patients, but it did partially increase AEs in all grades, with no discernible differences in serious AEs. Therefore, the combination treatment is not recommended in patients with advanced solid tumors.

**Systematic Review Registration:** https://www.crd.york.ac.uk/PROSPERO/display_record.php?RecordID=400841, identifier CRD42023400841

## 1 Introduction

The high morbidity and mortality rates associated with cancer have made it a critical social and public health issue, highlighting the pressing need to develop more effective therapies (Canadian Cancer Statistics, 2023; Siegel et al., 2023). Even though cancer mortality has steadily declined since 1991, 609,820 cancer deaths will occur by 2023 based on statistics from the American Cancer Society. There are a variety of cancer treatments exist, such as surgical intervention, radiation therapy, and systemic therapy comprising chemotherapy, targeted therapy, hormonal therapy, and immunotherapy (Miller et al., 2022). However, despite their efficacy, each cancer treatment modality has inherent limitations. Surgical intervention may be inadequate for certain solid tumors which are difficult to completely resect, and distant metastasis remains a challenge (Miller et al., 2022). Radiotherapy and traditional chemotherapy could result in damage to normal tissues while targeting tumor cells due to low selectivity (Schaue and McBride, 2015; Miller et al., 2022). Although promising for oncogene-driven cancers, targeted therapy benefits only a small subset of patients with specific tumor types (Zugazagoitia et al., 2016). Immune checkpoint protein expression levels in tumor cells place restrictions on immunotherapy outcomes (Zugazagoitia et al., 2016). What’s worse, immunotherapy may result in unfavorable immune-related side effects (Kennedy and Salama, 2020). In the past few years, there has been a rising curiosity in exploring novel treatment options for cancer that can overcome the limitations of current modalities. The application of oncolytic viruses is a promising strategy that has surfaced, encompassing the utilization of either naturally occurring or scientifically manipulated viruses that possess the capability of selectively reproducing and eradicating cancer cells (Sze et al., 2013; Harrington et al., 2019; Hemminki et al., 2020).

Pelareorep, also known as Reolysin^®^, is an isolate of an unmodified type 3 Dearing (T3D) reovirus, which has been widely studied as an anticancer agent of oncolytic virus therapy (Carew et al., 2013; Fukuhara et al., 2016; Gong et al., 2016; Noonan et al., 2016). Unlike various oncolytic viruses such as T-Vec, G47∆, JX-594, and CG0070, the pelareorep virus is unique because it is a naturally occurring virus and has long been used in cancer treatment, which could be traceable to the 1960s (Fukuhara et al., 2016). Furthermore, unlike other genetically engineered oncolytic viruses, it is not assumed to pose harm to humans (Fukuhara et al., 2016). Despite the existence of other reovirus serotypes, including Lang type 1, Jones type 2, and Abney type 3, the T3D strain remains the only naturally occurring therapeutic reovirus that is currently used for clinical purposes as a therapeutic agent (Norman and Lee, 2000). Several solid tumors and hematological cancers have shown promising results with this particular strain (Gong et al., 2016). Researchers have found that Pelareorep selectively replicates in tumor cells activating Ras signaling pathway while keeping normal cells unaffected (Coffey et al., 1998; Strong et al., 1998). H-Ras, K-Ras, and N-Ras mutations play an important role in human oncogenesis (Prior et al., 2012; Chen et al., 2019; Khan et al., 2019), and many types of human tumors have Ras mutations, consist of pancreatic, lung, colorectal, and thyroid cancer (Bos, 1989; Jiffry et al., 2021). As such, pelareorep represents a promising emerging therapy for these patients. It was approved to treat ovarian cancers, malignant gliomas, pancreatic cancers, metastatic breast cancers, and gastric cancers by the United States Food and Drug Administration (FDA) in 2015 and in subsequent years.

In a phase II single-arm clinical trial conducted in the United States, pelareorep combined with carboplatin and paclitaxel was found to be safe and well-tolerated for metastatic melanoma patients (Mahalingam et al., 2017). In addition, Mahalingam et al. (2018) carried out a single-arm study on advanced pancreatic adenocarcinoma patients and also found that pelareorep plus gemcitabine was well-tolerated, with a median PFS of 7.1 months and a median OS of 10.3 months. Afterwards, multiple randomized clinical trials indicated that pelareorep addition to chemotherapy did not provide any significant advantages when compared to chemotherapy alone. These results contradicted the findings of the single-arm studies conducted earlier. For instance, Eigl et al. (2018) reported that pelareorep did not improve PFS or ORR in patients with metastatic prostate cancer when given alongside chemotherapy. The randomized clinical trials conducted by Noonan et al. (2016), Bernstein et al. (2018), Cohn et al. (2017), and Bradbury et al. (2018) arrived at similar conclusions as well. However, the sample sizes of each trial were relatively small, which could produce controversial results. To enhance the credibility of the conclusion, here we sought to undertake a meta-analysis to evaluate the effectiveness and safety of pelareorep when accompanied by chemotherapy in randomized controlled trials. As far as we know, there is no meta-analysis focus on this topic.

Our study examined the effectiveness and safety of pelareorep in conjunction with various chemotherapeutic drugs, including carboplatin (Noonan et al., 2016), paclitaxel (Noonan et al., 2016; Cohn et al., 2017; Bernstein et al., 2018), pemetrexed (Bradbury et al., 2018), and docetaxel (Bradbury et al., 2018; Eigl et al., 2018). The study began with an assessment of the therapeutic efficacy of pelareorep, considering 1-year OS, 2-year OS, 4-month PFS, 1-year PFS, and ORR. A comprehensive evaluation of adverse events (AEs) was subsequently conducted, including all grades of AEs and grades ≥ 3 AEs. The findings of this meta-analysis have the potential to improve our comprehension of the safety and effectiveness of combining pelareorep with chemotherapy in randomized controlled trials, thereby informing future research and guiding clinical therapy.

## 2 Methods

### 2.1 Literature search strategy

We searched PubMed, EMBASE, Cochrane Library, and Web of Science (WOS) databases, covering studies published through 30 January 2023, using “Oncolytic viruses,” “Oncolytic virotherapy,” “Oncolytic reovirus,” “Pelareorep,” “Reolysin,” “Chemotherapy,” and “Cancer” as search terms, with no language restriction. Specific search strategies can be checked in Supplementary Material. This research was conducted according to the PRISMA guidelines (Page et al., 2021) and was pre-registered on PROSPERO (CRD42023400841).

### 2.2 Inclusion and exclusion criteria

We included studies that met specific criteria in our meta-analysis, for example: 1) randomized controlled trials (RCTs) involving cancer patients who received pelareorep as a treatment; 2) the studies reported on certain following outcomes: OS, PFS, ORR, or AEs; and 3) the control groups in the studies consisted of cancer patients who received the control regimen without pelareorep. The following exclusion criteria were applied: 1) conference abstracts, single-arm studies, clinical trial protocols, case reports, cohort studies, reviews, meta-analyses, and animal or *in vitro* studies were unselected; 2) studies where pelareorep therapy was used in the control group were excluded; 3) studies with overlapping or replicated data were also removed. Potentially eligible articles were screened by two independent investigators on the basis of titles and abstracts. Remaining records were evaluated in full text subsequently for eligibility. In cases where there were disagreements on the selection of studies, discussions with other investigators were employed.

### 2.3 Data extraction

Two researchers extracted data independently, and a third conducted a full review of every incorporated study to resolve discrepancies. The extracted data encompassed following items: first author, year, trial type, country, tumor type, total number, race, gender, age, treatment for the patients, administration of pelareorep and clinical outcome. The primary endpoints of this study were 1-year OS, 2-year OS, 4-month PFS, 1-year PFS, and ORR, while adverse events accessed according to Common Terminology Criteria for Adverse Events Version 4.0 (CTCAE 4.0) were considered as secondary endpoints. In addition, to avoid missing any relevant information, we scrutinized all the Supplementary Material of the included studies.

### 2.4 Quality assessment

The quality of studies was assessed based on the Cochrane Collaboration’s tool and the assessment results were classified as low-risk, high-risk, or unclear based on the available information (Higgins et al., 2011). The unclear risk category was considered when insufficient information was available. Any discrepancies were resolved through consensuses.

### 2.5 Statistical analysis

We adopted RevMan 5.3 and STATA 17.0 for statistical analysis in our study. The results were displayed as odds ratios (ORs) with 95% confidence intervals (CIs). To assess the heterogeneity among the randomized controlled trials included, the Chi-square test (Chi^2^), degree of freedom (df), and index of heterogeneity (I^2^) were performed. Random effect models were used when there was a difference in statistics in heterogeneity (I^2^ ≥ 50% or *p*-value < 0.05). Conversely, we employed a fixed effect model in the absence of significant heterogeneity (Chen and Benedetti, 2017). Besides, assessment of publication bias was done to all included studies by using Harbord test and Begg’s test (Lin and Chu, 2018). The criteria of statistically significant were *p*-value < 0.05.

## 3 Results

### 3.1 Study search and selection

**FIGURE 1:**
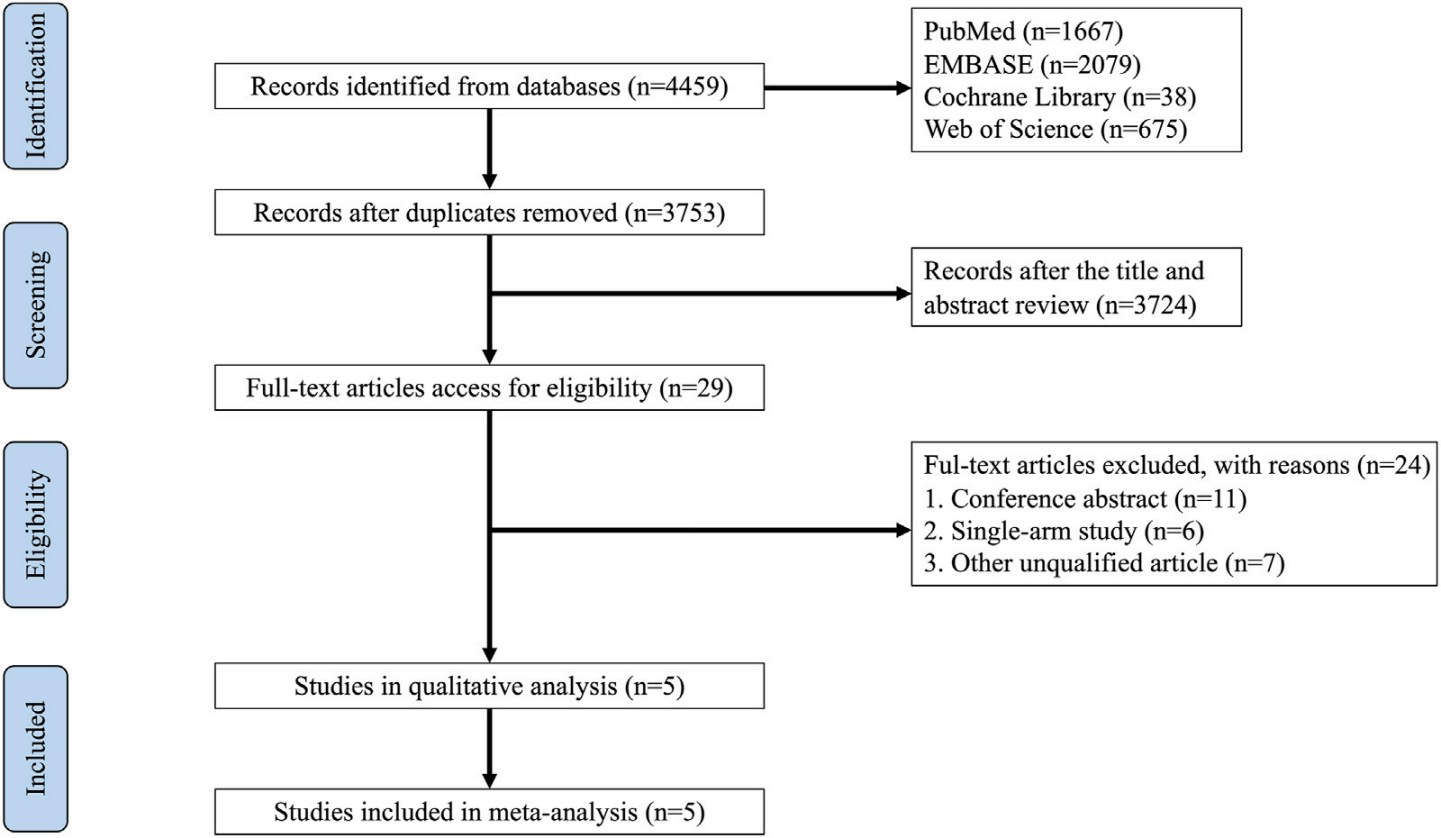
Flow chart of the study selection processes.

The databases of PubMed, EMBASE, Cochrane Library, and Web of Science (WOS) yielded 4,459 records in total. After removing duplicates by using EndNote 20, 3,753 records remained. Of these, 29 references were deemed eligible according to their titles and abstracts, and 24 studies were disregarded due to inappropriate contents, such as conference abstracts, single-arm studies, clinical trial protocols, and other non-qualified articles. Ultimately, 5 RCTs comprising 492 patients were selected for this meta-analysis. In Figure 1, a detailed illustration of how studies were selected is shown.

### 3.2 Characteristics of selected studies

This meta-analysis included 5 prospective phase Ⅱ clinical trials conducted in the United States of America and Canada, with a combined total of 492 patients. The studies were all published between June 2016 and June 2018 and involved patients of different races, including Caucasian, African American, white, and Canadian. Tables 1, 2 provide detailed information on the major features and outcomes of these studies. Cancer types comprising non-small-cell carcinoma, metastatic cancers such as breast cancer and castration-resistant prostate cancer, as well as recurrent cancers like ovarian cancer, tubal cancer and peritoneal cancer, were investigated in these included studies which evaluated the effectiveness and recorded the adverse events linked to the use of pelareorep in combination with chemotherapy.

**TABLE 1:** Major features of the included studies.

First author, year	Trial type	Country	Races	Tumor type	No. of patients	Gender (M/F)	Age (Years)	Treatment arms	Administration
Arnie, 2016	RCT, phase II	United States	Caucasian, African American	Metastatic pancreatic adenocarcinoma	73	41/32	EG: median 61.5 (39–84) CG: median 66 (45–81)	Pelareorep + carboplatin + paclitaxel vs. carboplatin + paclitaxel	Intravenous
Bernstein et al. (2018)	RCT, phase II	Canada	Canadian	Metastatic breast cancer	74	0/74	EG: median 61 (44–78) CG: median 57 (36–73)	Pelareorep + paclitaxel vs. paclitaxel	Intravenous
Cohn et al. (2017)	RCT, phase II	United States	White	Recurrent ovarian, tubal, or peritoneal cancer	108	0/108	No data	Pelareorep + paclitaxel vs. paclitaxel	Intravenous
Eigl et al. (2018)	RCT, phase II	Canada	Canadian	Metastatic castration resistant prostate cancer	85	85/0	EG: median 69.1 (50.3–83.7) CG: median 68.6 (49.7–86.6)	Pelareorep + docetaxel vs. docetaxel	Intravenous
Penelope, 2018	RCT, phase II	Canada	Canadian	Non-Small Cell Lung Cancer	152	77/75	EG: median 63.5 (23–78) CG: median 64.5 (39–84)	Pelareorep + pemetrexed/docetaxel vs. pemetrexed/docetaxel	Intravenous

EG, experimental group; CG, control group.

**TABLE 2:** Major outcomes of the included studies.

First author, year	Median OS (Months)	HR (95%CI) for OS	Median PFS (Months)	HR (95%CI) for PFS	ORR
Anne, 2016	EG: 7.3	1.12 (0.67, 1.87)	EG: 4.9	0.88 (0.54, 1.42)	EG: 7
	CG: 8.8		CG: 5.2		CG: 7
Bernstein et al. (2018)	EG: 17.4	0.65 (0.39, 1.09)	EG: 3.78	1.04 (0.64, 1.69)	EG: 9
	CG: 10.4		CG: 3.38		CG: 9
Eigl et al. (2018)	EG: 12.6	1.01 (0.64, 1.58)	EG: 4.4	1.11 (0.73, 1.70)	EG: 8
	CG: 13.1		CG: 4.3		CG: 9
Eigl et al. (2018)	EG: 19.1	1.83 (0.96, 3.52)	No data	No data	EG: 11
	CG: 21.1				CG: 14
Penelope, 2018	EG: 7.8	0.98 (0.68, 1.40)	EG: 3.0	0.90 (0.65, 1.25)	EG: 11
	CG: 7.4		CG: 2.8		CG: 11

OS, overall survival; PFS, progression-free survival; ORR, objective response rate; HR, hazard ratio; CI, confidence interval; EG, experimental group; CG, control group.

### 3.3 Quality assessment

**FIGURE 2:**
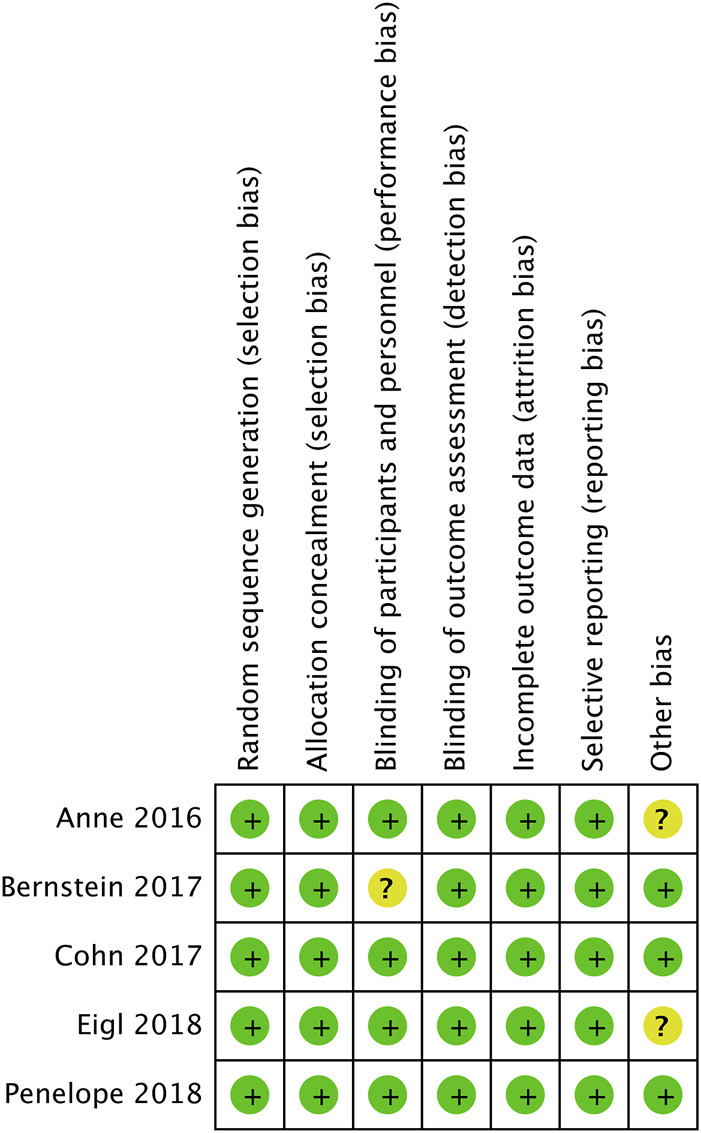
Evaluation of the risk of bias for the included studies.

Figure 2 shows evaluation of bias risk for 5 included RCTs. In every trial, random allocation and sequence generation were employed. Although all trials were open-label, no blind methods had little influence on quality evaluation of literatures. Therefore, it was deemed to be low risk.

### 3.4 Efficacy

#### 3.4.1 Overall survival (OS)

**FIGURE 3:**
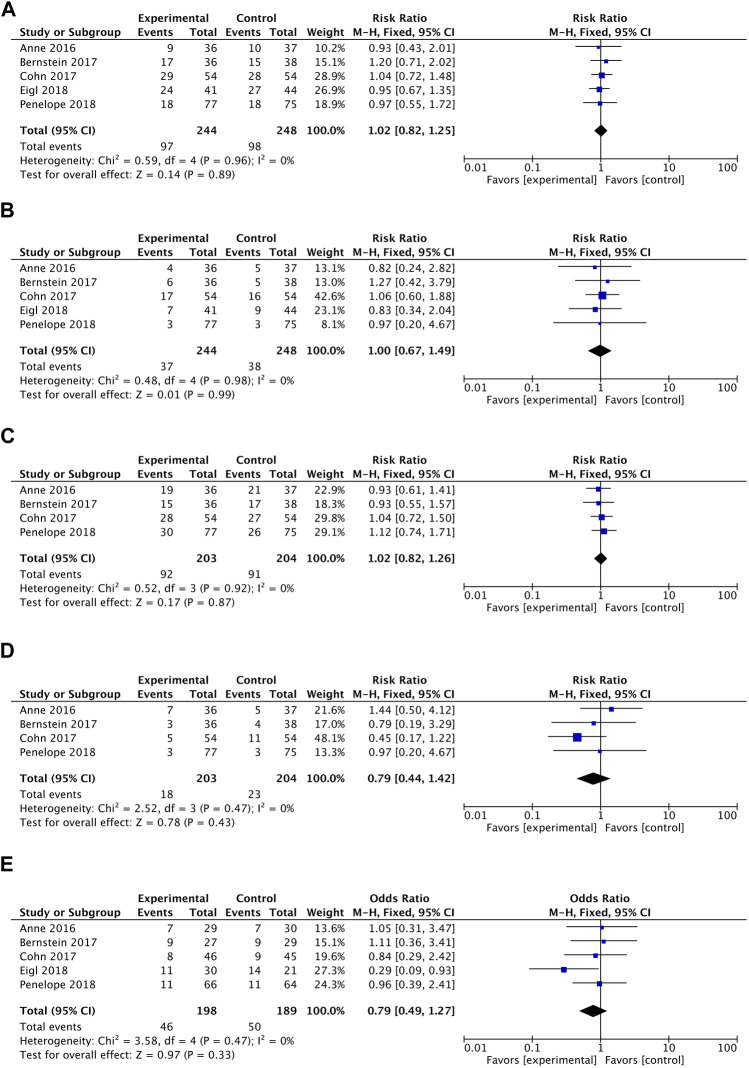
**(A)** Forest plot presenting the combined risk ratios for 1-year OS. **(B)** Forest plot presenting the combined risk ratios for 2-year OS. **(C)** Forest plot presenting the combined risk ratios for 4-month PFS. **(D)** Forest plot presenting the combined risk ratios for 1-year PFS. **(E)** Forest plot presenting the combined risk ratios for ORR.

**FIGURE 4:**
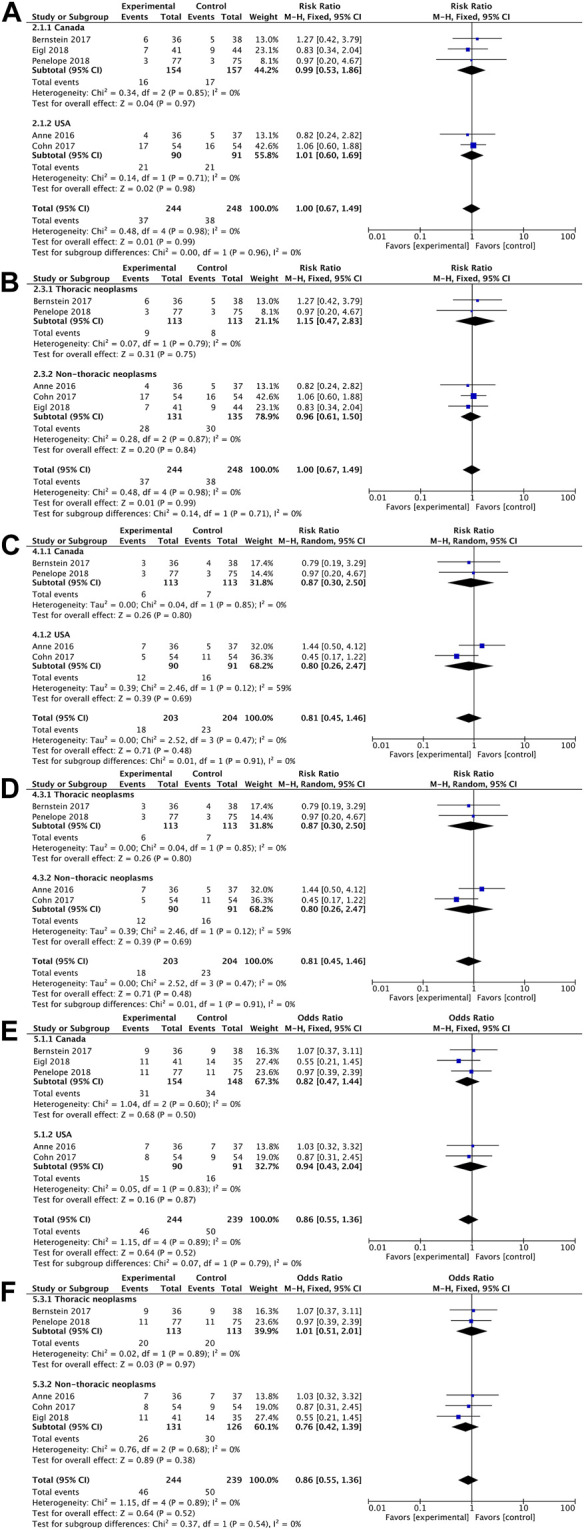
**(A)** Forest plot presenting the combined risk ratios for 2-year OS in different countries; **(B)** Forest plot presenting the combined risk ratios for 2-year OS in different tumor sites; **(C)** Forest plot presenting the combined risk ratios for 1-year PFS in different countries; **(D)** Forest plot presenting the combined risk ratios for 1-year PFS in different tumor sites; **(E)** Forest plot presenting the combined risk ratios for ORR in different countries; **(F)** Forest plot presenting the combined risk ratios for ORR in different tumor sites.

In terms of overall survival rate, pelareorep plus chemotherapy in advanced solid tumor patients did not demonstrate any significant improvements compared to chemotherapy alone. An analysis of five studies showed no statistical differences between pelareoreps and controls at 1-year OS [RR = 1.02, 95%CI = (0.82–1.25)] (Figure 3A). Similarly, adding pelareorep did not lead to any significant differences in 2-year OS based on the analysis of five studies [RR = 1.00, 95%CI = (0.67–1.49)] (Figure 3B). The analysis of 2-year OS by subgroups indicated that the patients from both Canada [RR = 0.99, 95%CI = (0.53–1.86)] and the United States [RR = 1.01, 95%CI = (0.60–1.69)] (Figure 4A) did not benefit from pelareorep. Furthermore, no statistical significances were found in the analysis of pelareorep’s effect on tumors specific to the thorax [R = 1.15, 95%CI = (0.47–2.83)] or other parts of the body [RR = 0.96, 95%CI = (0.61, 1.50)] (Figure 4B).

#### 3.4.2 Progression-free survival (PFS)

Except for the study conducted by Eigl et al., four studies presented data on progression-free survival rates. Pelareorep when combined with chemotherapy did not significantly improve 4-month PFS [RR = 1.02, 95%CI = (0.82–1.26)] (Figure 3C) or 1-year PFS [RR = 0.79, 95%CI = (0.44–1.42)] (Figure 3D). The subgroup analysis of 1-year PFS did not yield statistically significant results for pelareorep in either Canada [RR = 0.87, 95%CI = (0.30–2.50)] or the United States [RR = 0.80, 95%CI = (0.26–2.47)] (Figure 4C). Similarly, there were no significant differences in either thoracic [RR = 0.87, 95%CI = (0.30–2.50)] or non-thoracic tumors [RR = 0.80, 95%CI = (0.26–2.47)] (Figure 4D).

#### 3.4.3 Objective response rate (ORR)

All the five studies reported ORR. Between the groups receiving chemotherapy alone and those receiving both chemotherapy and pelareorep, the ORR was not statistically different [OR = 0.79, 95%CI = (0.49–1.27)] (Figure 3E). Subgroup analysis of ORR did not show significantly better results for pelareorep than chemotherapy alone in either Canada [OR = 0.82, 95%CI = (0.47–1.44)] or the United States [OR = 0.94, 95%CI = (0.43–2.04)] (Figure 4E). In the other subgroup, we found a similar result. There were no significant differences in ORR for either thoracic tumors [OR = 1.01, 95%CI = (0.51–2.01)] or non-thoracic tumors [OR = 0.76, 95% CI = (0.42–1.39)] (Figure 4F).

#### 3.4.4 Safety

Our research involved examining both adverse events (AEs) of all grades and severe adverse events (with a grade of ≥ 3). Patients receiving pelareorep were more likely to develop fever [RR = 3.10, 95%CI = (1.48–6.52)], nausea [RR = 1.19, 95%CI = (1.02–1.38)], diarrhea [RR = 1.87, 95%CI = (1.39–2.52)], chills [RR = 4.14, 95%CI = (2.30–7.43)], headache [RR = 1.46, 95%CI = (1.02–2.09)], vomiting [RR = 1.38, 95%CI = (1.06–1.80)] and flu-like symptoms [RR = 4.18, 95%CI = (2.19–7.98)] (Table 3). However, when pelareorep was combined with chemotherapy, severe adverse events did not differ statistically from chemotherapy alone (Table 4).

**TABLE 3:** Any-grade adverse events with the incidence of experimental group (EG).

Adverse events	*I* ^2^ (%)	RR (95%CI)	*p*	Incidence of EG (%)	Adverse events	*I* ^2^ (%)	RR (95%CI)	*p*	Incidence of EG (%)
Fatigue	26	1.00 (0.92, 1.09)	1.00	87.58	Fever	70	3.10 (1.48, 6.52)	0.003*	55.90
Dyspnea	54	1.21 (0.81, 1.81)	0.36	54.66	Alopecia	21	1.01 (0.83, 1.23)	0.91	52.17
Nausea	26	1.19 (1.02, 1.38)	0.02*	68.08	Peripheral sensory neuropathy	59	0.83 (0.59, 1.15)	0.26	38.97
Anorexia	0	1.17 (0.95, 1.45)	0.14	55.90	Diarrhea	0	1.87 (1.39, 2.52)	<0.0001*	49.07
Constipation	0	0.99 (0.79, 1.24)	0.92	47.83	Insomnia	0	1.27 (0.95, 1.70)	0.11	41.61
Chills	0	4.14 (2.30, 7.43)	<0.00001*	32.30	Headache	2	1.46 (1.02, 2.09)	0.04*	34.16
Cough	34	0.83 (0.58, 1.19)	0.30	37.89	Myalgia	0	1.30 (0.88, 1.90)	0.19	28.57
Vomiting	0	1.38 (1.06, 1.80)	0.02*	40.85	Flu-like symptoms	0	4.18 (2.19, 7.98)	<0.0001*	27.33
Mucositis oral	0	1.11 (0.80, 1.54)	0.53	30.43	Back pain	0	0.95 (0.69, 1.31)	0.77	31.06
Maculopapular rash	50	1.37 (0.50, 3.80)	0.54	13.04	Dizziness	32	1.22 (0.77, 1.94)	0.40	20.50
Edema limbs	0	0.91 (0.63, 1.31)	0.61	23.60	Anxiety	0	0.88 (0.52, 1.50)	0.64	13.66
Arthralgia	36	1.04 (0.66, 1.64)	0.86	19.25	Extremity pain	74	0.91 (0.39, 2.08)	0.81	25.47

*Statistically significant value, *I* ^2^, index of heterogeneity; RR, risk ratio; CI, confidence interval.

**TABLE 4:** Adverse events in grade ≥ 3 with the incidence of experimental group (EG).

Adverse events	*I* ^2^ (%)	RR (95%CI)	*p*	Incidence of EG (%)	Adverse events	*I* ^2^ (%)	RR (95%CI)	*p*	Incidence of EG (%)
Fatigue	0	1.31 (0.82, 2.08)	0.26	17.77	Fever	0	1.40 (0.24, 8.18)	0.71	2.50
Nausea	2	0.94 (0.38, 2.36)	0.90	3.76	Peripheral sensory neuropathy	0	0.49 (0.13, 1.88)	0.30	1.47
Anorexia	11	1.28 (0.25, 6.46)	0.76	2.50	Diarrhea	54	1.41 (0.18, 11.38)	0.74	4.97
Vomiting	22	0.38 (0.11, 1.30)	0.12	1.74	Flu-like symptoms	0	2.79 (0.29, 26.37)	0.37	1.67
Infection	0	1.15 (0.61, 2.15)	0.67	11.52	Febrile neutropenia	37	1.24 (0.77, 2.00)	0.37	20.13
Neutropenia	80	1.49 (0.48, 4.66)	0.49	39.77	Leukopenia	90	1.84 (0.23, 14.36)	0.56	32.95
Thrombocytopenia	0	1.02 (0.51, 2.05)	0.96	9.30	Anemia	15	1.23 (0.73, 2.09)	0.44	19.38

*I* ^2^, index of heterogeneity; RR, risk ratio; CI, confidence interval.

#### 3.4.5 Publication bias analysis and sensitivity analysis

Since our meta-analysis included fewer than 10 studies, it was not appropriate to use funnel plots to evaluate publication bias (Lau et al., 2006). Therefore, we used the Harbord test and Begg’s tests rather than Egger’s and funnel plots for publication bias assessment. 1-year OS (*p* = 0.9612 in Harbord test and *p* = 1.0000 in Begg’s test), 2-year OS (*p* = 0.8615 in Harbord test and *p* = 1.0000 in Begg’s test), 4-month PFS (*p* = 0.7377 in Harbord test and *p* = 1.0000 in Begg’s test), 1-year PFS (*p* = 0.5667 in Harbord test and *p* = 0.7341 in Begg’s test), ORR (*p* = 0.4067 in Harbord test and *p* = 0.4624 in Begg’s test) did not find publication bias. Additionally, we also performed sensitivity analyses by omitting each study individually in order to estimate their effects on the overall results (Supplementary Material). The results indicated that the exclusion of any individual study had minimal impact on the overall conclusion. Hence, publication bias had little effect on the correctness of our deductions.

## 4 Discussion

In this meta-analysis, we included five RCTs with the aim of comparing the efficacy and safety of pelareorep plus chemotherapy versus chemotherapy alone in treating different types of advanced solid tumors. Our analysis focused on five parameters, namely, 1-year OS, 2-year OS, 4-month PFS, 1-year PFS, and ORR. We also conducted a subgroup analysis based on countries and tumor sites. The results demonstrated that 1-year OS, 2-year OS, 4-month PFS, 1-year PFS, and ORR in two treatment groups were not statistically different. These findings were consistent across subgroups, thereby indicating that combining pelareorep with traditional chemotherapy did not enhance the efficacy of treatment or increase survival rates for patients with advanced solid tumors. It is worth noting that our results contradict the findings of previous single-arm clinical trials investigating the use of pelareorep in combination with chemotherapeutic agents (Karapanagiotou et al., 2012; Mahalingam et al., 2017, 2018). The absence of internal controls in single-arm studies poses significant confounding factors, leading to potential bias and misinterpretation of results, which will weaken the effectiveness of any inferences drawn from the intervention’s impact. As a result, we specifically chose randomized controlled trials that had more conclusive results to strengthen the persuasiveness of our meta-analysis.

Several meta-analyses have already confirmed that combining oncolytic viruses with chemotherapy is effective and safe for patients with tumors (Li Z. et al., 2020; Li et al., 2020 Y.; Liu et al., 2022). Nonetheless, there are still debates on whether patients with advanced solid tumors can benefit from the treatment of pelareorep combined with palliative chemotherapy. Various oncolytic viruses including T-Vec, G47∆, JX-594, CG0070, and Reolysin are currently being clinically developed (Fukuhara et al., 2016). Of these, Reolysin stands out as a naturally existing virus with a lengthy history of cancer treatment, and it is not believed to be harmful to humans, unlike other oncolytic viruses created through genetic manipulation. The replication of Pelareorep specific to tumor cells based on Ras-mutated proteins, leading to the disruption of tumor cells through an oncolytic effect (Sze et al., 2013; Gong and Mita, 2014; Gong et al., 2016). As the virus is released from the infected tumor cells, which is more infections than the original virus, leading to more effective apoptosis of adjacent tumor cells (Marcato et al., 2007). Innate as well as adaptive antitumor immune reactions induced by changes in tumor microenvironments take an important position in cell death. For one thing, the reovirus can activate dendritic cells directly, which subsequently promotes the generation and affection of inflammatory cytokines, nature killer (NK) cells, and T cells (Prestwich et al., 2009), and the inflammatory cytokines or chemokines released from the infective tumor cells could launch an immune response (Errington et al., 2008). For another thing, pelareorep could trigger apoptosis of tumor cells by promoting heightened endoplasmic reticular stress (Carew et al., 2013). In addition to necrosis and apoptosis, autophagy was also an essential cell death pathway induced by pelareorep in colorectal cancer cells (Jiffry et al., 2021) and multiple myeloma cells (Thirukkumaran et al., 2013).

There are two methods of administration of pelareorep in previous research basically. One is intratumoral, while the other is intravenous. It was reported that the intratumoral injection of pelareorep yielded a good therapeutic outcome in experimental mice (Jiffry et al., 2021). Analogously, a meta-analysis performed by Li and colleagues showed significantly higher ORR in patients with intratumoral injection of oncolytic viruses when compared to the patients with intravenous administration (Li Z. et al., 2020). We assume that in contrast to intratumoral injection, the intravenous (IV) administration of pelareorep has a slower onset and lower specificity. All of the included RCTs administered pelareorep via IV injection, which may be attributed to the following reasons: 1) to maintain consistency with the administration of traditional chemotherapeutic agents; and 2) most patients included in these RCTs had advanced or metastatic cancer and had failed previous treatments, making it difficult to cover all lesions with intratumoral injection. However, IV administration may result in viremia, which could make pelareorep neutralized by antibodies in patients who have been exposed to reovirus or vaccination before, consequently leading to lower treatment efficacy than expected (Jackson and Muldoon, 1973; Fukuhara et al., 2016). Moreover, IV infusion of pelareorep could increase the incidence of systemic adverse reactions, thereby increasing the risk of treatment interruption, which will be discussed later in this section. Therefore, it may be beneficial to consider improving the administration method of pelareorep or appropriately increasing the dosage within the tolerable range for patients to enhance the viral load at the lesion site and better exert the anti-tumor effect of pelareorep.

In general, pelareorep is considered well-tolerated in most patients (Errington et al., 2008; Prestwich et al., 2009). Combining pelareorep with conventional chemotherapy has not been shown to produce a synergistic effect on adverse events, resulting in a higher incidence of AEs. Nonetheless, this meta-analysis has shown that the incidence of certain adverse events remains elevated when pelareorep is administered along with chemotherapy. Any-grade adverse events with an incidence rate ≥ 10% included fatigue (87.58%), fever (55.90%), dyspnea (54.66%), alopecia (52.17%), nausea (68.08%), peripheral sensory neuropathy (38.97%), anorexia (55.90%), diarrhea (49.07%), constipation (47.83%), insomnia (41.61%), chills (32.30%), headache (34.16%), cough (37.89%), myalgia (28.57%), vomiting (40.85%), flu-like symptoms (27.33%), mucositis oral (30.43%), back pain (31.06%), maculopapular rash (13.04%), dizziness (20.05%), edema limbs (23.60%), anxiety (13.66%), arthralgia (19.25%), and extremity pain (25.47%). Severe adverse events with an incidence rate ≥ 5% included fatigue (17.77%), infection (11.52%), febrile neutropenia (20.13%), neutropenia (39.77%), leukopenia (32.95%), thrombocytopenia (9.30%), and anemia (19.38%). Although our study did not show that pelareorep combined with chemotherapy would increase the occurrence of severe adverse events, caution should still be exercised when using pelareorep in patients who may be vulnerable to adverse effects. In such cases, particular treatment measures should be taken to mitigate any potential detrimental effects.

Our meta-analysis has several drawbacks. Firstly, our strict inclusion criteria resulted in the identification of only five RCTs, which led to a relatively small study sample size. Additionally, some of the RCTs failed to report data on all adverse events during the analysis, which may have influenced the strength of our conclusions. Furthermore, pelareorep has only been studied in a few phase III trials, and even fewer studies combining pelareorep with conventional chemotherapy. As a result, the articles included in our meta-analysis involved various cancers with varying baseline patient characteristics, making it difficult to compare the data. Despite similar treatment methods, some indicators varied widely between studies, resulting in substantial differences in results when all data were compared.

Despite these limitations, it is still essential to discuss whether pelareorep can benefit cancer patients for its unique anti-tumor mechanism. Regrettably, our research show that combination therapy failed to improve treatment efficacy.

## 5 Conclusion

Our study compared the efficacy and safety of pelareorep plus chemotherapy with chemotherapy alone in advanced solid tumors. OS, PFS, ORR and AEs were all evaluated as endpoints in this study. The outcomes showed that combining pelareorep and chemotherapy did not increase efficacy but did increase the occurrence of adverse events, which means the combination therapy is not recommended.

## Data Availability

The original contributions presented in the study are included in the article/Supplementary Material, further inquiries can be directed to the corresponding author.
