# Supplementary material for: Effectiveness and safety of pelareorep plus chemotherapy versus chemotherapy alone for advanced solid tumors: a meta-analysis
Source: Front Pharmacol. 2023 Sep 26;14:1228225. doi: 10.3389/fphar.2023.1228225 (PMC10566296; doi:10.3389/fphar.2023.1228225)
Supplement: Supplementary file 1 [file Table1.docx]

Supplementary Material

Effectiveness and safety of pelareorep plus chemotherapy versus chemotherapy alone for advanced solid tumors: a meta-analysis

Renxian Xie, Hongxin Huang, Tong Chen, Xuehan Huang, Chuangzhen Chen*

*** Correspondence:** Corresponding Author: [czchen2@stu.edu.cn](mailto:czchen2@stu.edu.cn)

# Supplementary Figure


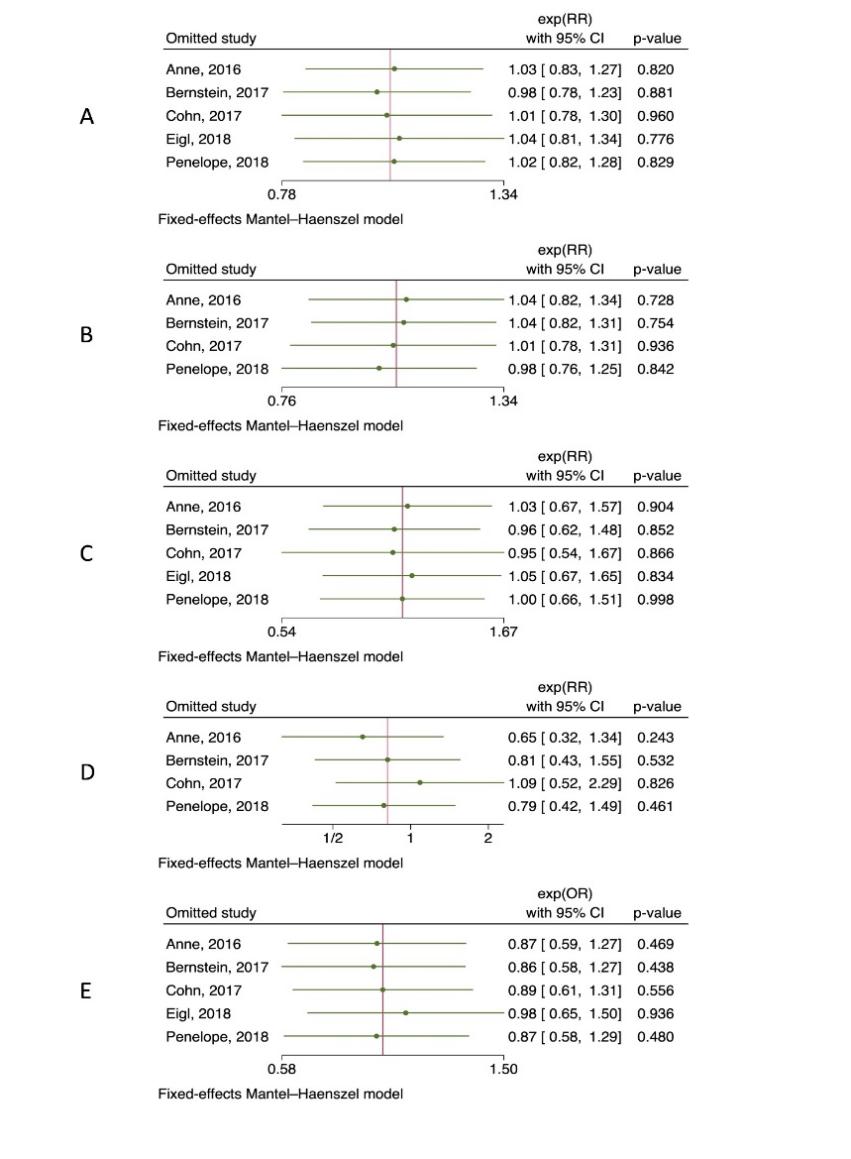


**Supplementary Figures S1A-E.** (A) Sensitivity analysis of 1-year OS. (B) Sensitivity analysis of 1-year PFS. (C) Sensitivity analysis of 2-year OS. (D) Sensitivity analysis of 4-month PFS. (E) Sensitivity analysis of ORR.

# Search Strategy

**PubMed**

((("Oncolytic Viruses"[MeSH Terms]) OR ("Oncolytic Virus"[Title/Abstract]) OR ("Virus, Oncolytic"[Title/Abstract]) OR ("Viruses, Oncolytic"[Title/Abstract]) OR ("Oncolytic Virotherapy"[MeSH Terms]) OR ("Oncolytic Virotherapies"[Title/Abstract]) OR ("Virotherapies, Oncolytic"[Title/Abstract]) OR ("Virotherapy, Oncolytic"[Title/Abstract]) OR ("Oncolytic Virus Therapy"[Title/Abstract]) OR ("Oncolytic Virus Therapies"[Title/Abstract]) OR ("Therapies, Oncolytic Virus"[Title/Abstract]) OR ("Therapy, Oncolytic Virus"[Title/Abstract]) OR ("Virus Therapies, Oncolytic"[Title/Abstract]) OR ("Virus Therapy, Oncolytic"[Title/Abstract]) OR ("Pelareorep"[Title/Abstract]) OR ("Reolysin"[Title/Abstract]) OR ("Reovirus type 3 Dearing"[Title/Abstract]) OR ("AN1004"[Title/Abstract]) OR ("oncolytic reovirus"[Title/Abstract])) AND (("Neoplasms"[Title/Abstract]) OR ("Tumor"[Title/Abstract]) OR ("Neoplasm"[Title/Abstract]) OR ("Tumors"[Title/Abstract]) OR ("Neoplasia"[Title/Abstract]) OR ("Neoplasias"[Title/Abstract]) OR ("Cancer"[Title/Abstract]) OR ("Cancers"[Title/Abstract]) OR ("Malignant Neoplasm"[Title/Abstract]) OR ("Malignancy"[Title/Abstract]) OR ("Malignancies"[Title/Abstract]) OR ("Malignant Neoplasms"[Title/Abstract]) OR ("Neoplasm, Malignant"[Title/Abstract]) OR ("Neoplasms, Malignant"[Title/Abstract])) AND ((chemotherapy) OR (chemotherapies)))

**Web of Science**

TOPIC: ("Oncolytic Virus" OR "Oncolytic Virotherapy" OR "Oncolytic Virotherapies" OR "Oncolytic Virus Therapies" OR "Reolysin" OR "Pelareorep" OR "Reovirus type 3 Dearing" OR "AN1004" OR "oncolytic reovirus") AND TOPIC: ("Neoplasm" OR "Tumor" OR "Neoplasia" OR "Cancer" OR "Malignancy" OR "Malignant Neoplasm" OR "Malignancies") AND All Text:("chemotherapy" OR "chemotherapies")

**Cochrane Library**

Title abstract keyword:("Oncolytic Virus" OR "Oncolytic Virotherapy" OR "Oncolytic Virotherapies" OR "Oncolytic Virus Therapies" OR "Reolysin" OR "Pelareorep" OR "Reovirus type 3 Dearing" OR "AN1004" OR "oncolytic reovirus") AND Title abstract keyword:("Neoplasm" OR "Tumor" OR "Neoplasia" OR "Cancer" OR "Malignancy" OR "Malignant Neoplasm" OR "Malignancies") AND All Text:("chemotherapy" OR "chemotherapies")

**EMBASE**

(('oncolytic reovirus'/exp OR 'pelareorep'/exp OR 'oncolytic virus'/exp OR 'oncolytic virotherapy'/exp) OR ('reolysin':ti,ab,kw OR 'reovirus type 3 dearing':ti,ab,kw OR 'an1004':ti,ab,kw)) AND ('cancer'/exp OR 'cancers'/exp OR 'malignant neoplasia'/exp OR 'malignant neoplastic disease'/exp OR 'malignant tumor'/exp OR 'malignant tumour'/exp OR 'neoplasia, malignant'/exp OR 'neoplasmic malignancy'/exp OR 'neoplastic malignancy'/exp OR 'oncologic malignancy'/exp OR 'oncological malignancy'/exp OR 'tumor, malignant'/exp OR 'tumoral malignancy'/exp OR 'tumorous malignancy'/exp OR 'tumour, malignant'/exp OR 'malignant neoplasm'/exp) AND ('chemotherapeutics'/exp OR 'chemotherapy'/exp)
